# Supplementary material for: Poverty induced inequality in nutrition among children born during 2010–2021 in India
Source: PLoS One. 2024 Nov 14;19(11):e0313596. doi: 10.1371/journal.pone.0313596 (PMC11563417; doi:10.1371/journal.pone.0313596)
Supplement: S1 Appendix — (PDF) [file pone.0313596.s001.pdf]

S1 Appendix: Sub-population-specific patterns in the HAZ scores of under-five children in India, NFHS, 2019-21

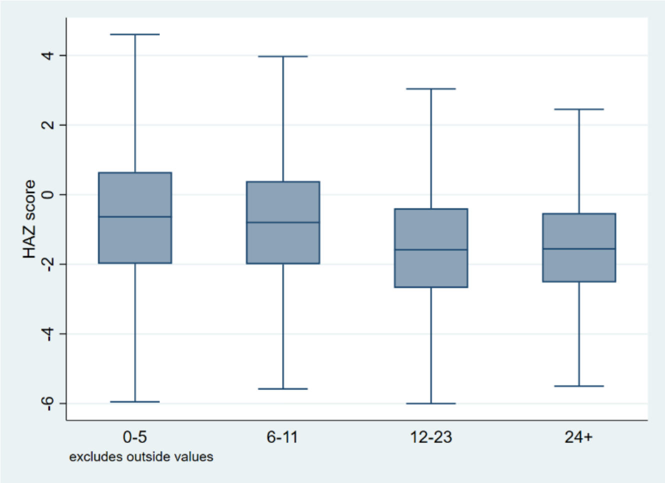

Fig 1. Box plot of HAZ score by age of the child, NFHS, 2019-21, India

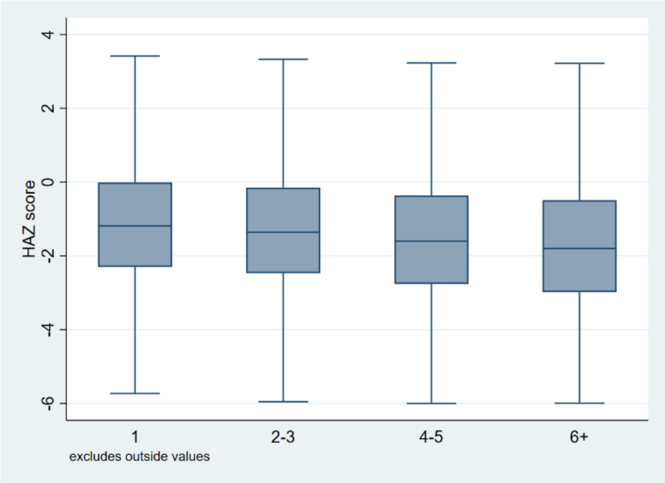

Fig 2. Box plot of HAZ score by birth order of the child, NFHS, 2019-21, India

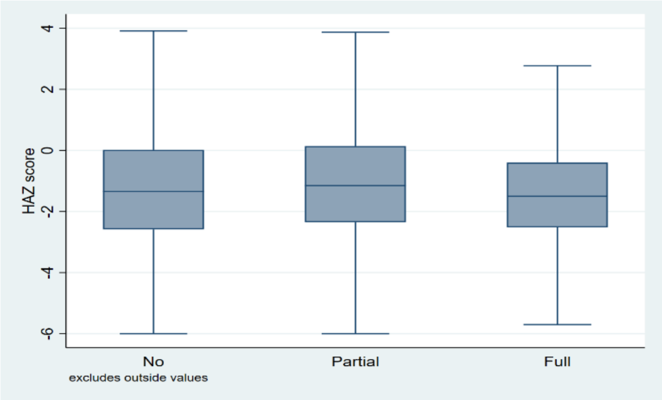

Fig 3. Box plot of HAZ score by immunization status of the child, NFHS, 2019-21, India

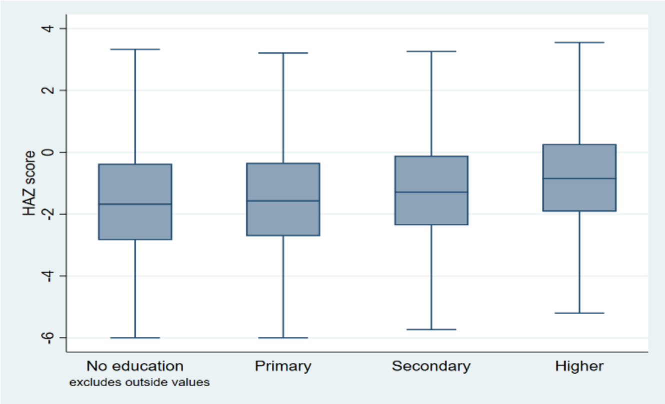

Fig 4. Box plot of HAZ score by mother's education of the child, NFHS, 2019-21, India

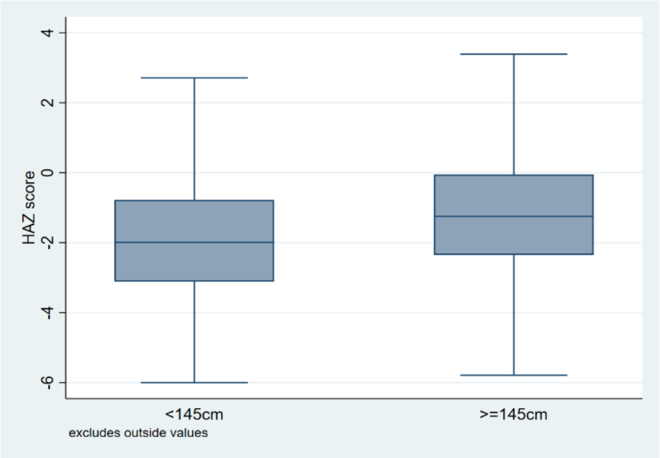

Fig 5. Box plot of HAZ score by mother's height of the child, NFHS, 2019-21, India

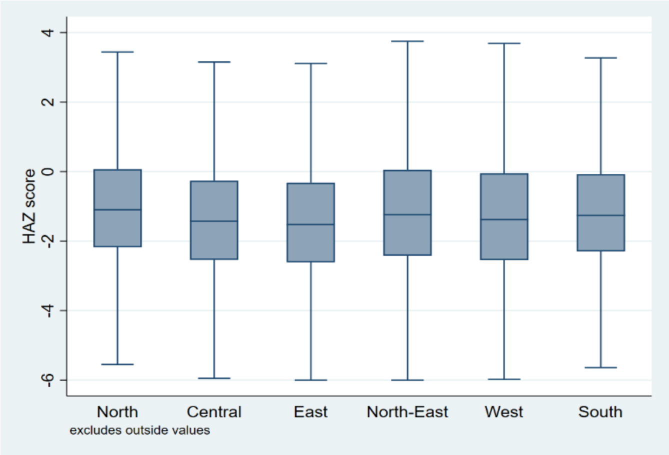

Fig 6. Box plot of HAZ score across regions, NFHS, 2019-21, India

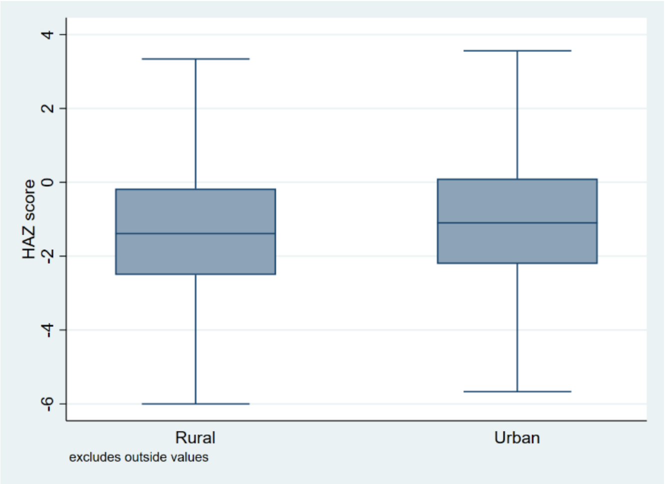

Fig 7. Box plot of HAZ score by place of residence, NFHS, 2019-21, India

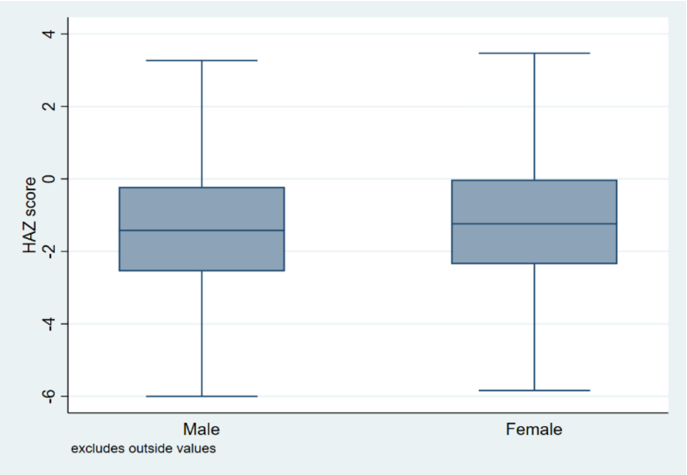

Fig 8. Box plot of HAZ score by gender of the child, NFHS, 2019-21, India

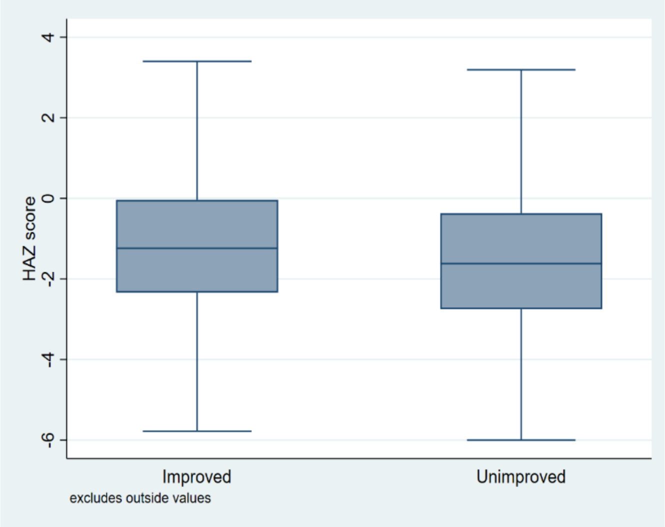

Fig 9. Box plot of HAZ score by sanitation facility, NFHS, 2019-21, India

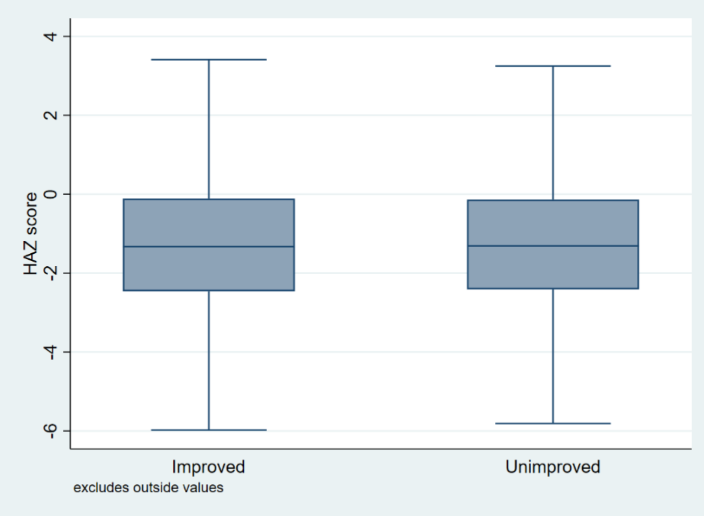

Fig 10. Box plot of HAZ score by source of drinking water, NFHS, 2019-21, India

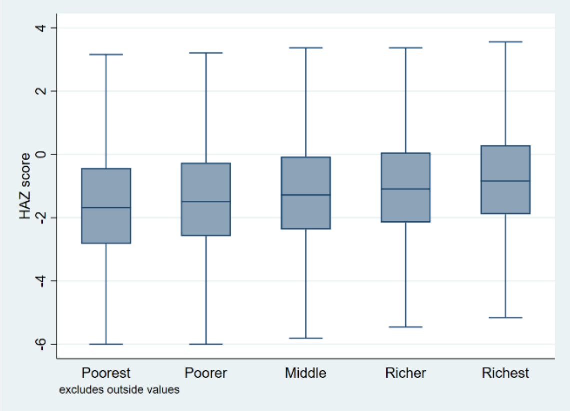

Fig 11. Box plot of HAZ score by wealth quintile, NFHS, 2019-21, India
