# Supplementary material for: Poverty induced inequality in nutrition among children born during 2010–2021 in India
Source: PLoS One. 2024 Nov 14;19(11):e0313596. doi: 10.1371/journal.pone.0313596 (PMC11563417; doi:10.1371/journal.pone.0313596)
Supplement: Attachment — Submitted filename: Reviewed PONE-D-21-08355_Reply.pdf [file pone.0313596.s003.pdf]

PLOS ONE

The extent of wealth poverty induced inequality in nutrition among children born during 2010-16 in India

--Manuscript Draft--

**Manuscript Number:**	PONE-D-21-08355
**Article Type:**	Research Article
**Full Title:**	The extent of wealth poverty induced inequality in nutrition among children born during 2010-16 in India
**Short Title:**	Wealth poverty induced inequality in child nutrition in India
**Corresponding Author:**	Junaid Khan, M.Phil<br>International Institute for Population Sciences<br>Mumbai, Maharashtra INDIA
**Keywords:**	Poverty, Stunting, Nutritional Inequality, Concentration Index, Sustainable Development Goals, National Family Health Survey, India
**Abstract:**	Introduction<br>Almost two-fifth of the children in India is stunted and among various factors, poverty gap in child nutrition is largest. Using the latest population based survey of National Family Health Survey, 2015-16 this paper examined the economic inequality in child stunting in India across the sub-populations and across states.<br>Methods<br>A sample of 225,002 children aged between 0-5 years from 568,200 households constitutes the study sample. The wealth index is used as the proxy of household's economic wellbeing and height-for-age (HAZ) z-score of a child is used to identify the stunting status of the child. Box plots were drawn to understand the distributional characteristics of the HAZ score. We calculated the Erreygers' corrected concentration index and decomposed the concentration indices using Gonzalo-Almorox and Urbanos-Garrido method.<br><br>Results<br>About half of the children from the poorest wealth quintile were stunted (51%), compared to 22% among the children from richest wealth quintile. Stunting was as high as 57% among the children of mothers with low stature (height less than 145 cm). Among various factors, the concentration index of stunting was observed highest among the children of 36-47 months (-0.28) followed by children of age 48-59 months (-0.27) and among the fully immunized children (-0.25). Factors like child age, birth order and sanitation showed positive elasticity. Due to uneven distribution of wealth, mother's education as a determinant of child stunting solely explained 33% of the overall inequality followed by improved access to sanitation (24%), mother's height (8%) and place of residence (5%).<br>Conclusions<br>In India, poverty differential in child malnutrition is acute among the different sub-population of children. And the concentration of stunting is higher among children of different background characteristics with higher wealth poverty. The social, demographic and household characteristics such as mother's education, improved sanitation and mother's height explained larger variation in overall inequality in child nutrition across India.
**Order of Authors:**	Junaid Khan, M.Phil
	Sanjay K Mohanty
**Additional Information:**	
**Question**	**Response**
**Financial Disclosure**<br><br>Enter a financial disclosure statement that describes the sources of funding for the	The author received no specific funding for this work.

Powered by Editorial Manager® and ProduXion Manager® from Aries Systems Corporation

work included in this submission. Review the submission guidelines for detailed requirements. View published research articles from *PLOS ONE* for specific examples.<br><br>This statement is required for submission and **will appear in the published article** if the submission is accepted. Please make sure it is accurate.<br><br>**Unfunded studies**<br>Enter: *The author(s) received no specific funding for this work.*<br><br>**Funded studies**<br>Enter a statement with the following details:<br>• Initials of the authors who received each award<br>• Grant numbers awarded to each author<br>• The full name of each funder<br>• URL of each funder website<br>• Did the sponsors or funders play any role in the study design, data collection and analysis, decision to publish, or preparation of the manuscript?<br>• **NO** - Include this sentence at the end of your statement: *The funders had no role in study design, data collection and analysis, decision to publish, or preparation of the manuscript.*<br>• **YES** - Specify the role(s) played.<br><br>* typeset	
**Competing Interests**<br><br>Use the instructions below to enter a competing interest statement for this submission. On behalf of all authors, disclose any competing interests that could be perceived to bias this work—acknowledging all financial support and any other relevant financial or non-financial competing interests.<br><br>This statement is required for submission and **will appear in the published article** if the submission is accepted. Please make sure it is accurate and that any funding sources listed in your Funding Information	The authors have declared that no competing interests exist.

later in the submission form are also declared in your Financial Disclosure statement.<br><br>View published research articles from *PLOS ONE* for specific examples.<br><br>**NO authors have competing interests**<br><br>Enter: *The authors have declared that no competing interests exist.*<br><br>**Authors with competing interests**<br><br>Enter competing interest details beginning with this statement:<br><br>*I have read the journal's policy and the authors of this manuscript have the following competing interests: [insert competing interests here]*<br><br>* typeset	
**Ethics Statement**<br><br>Enter an ethics statement for this submission. This statement is required if the study involved:<br><br>• Human participants<br>• Human specimens or tissue<br>• Vertebrate animals or cephalopods<br>• Vertebrate embryos or tissues<br>• Field research<br><br>Write "N/A" if the submission does not require an ethics statement.<br><br>General guidance is provided below. Consult the submission guidelines for detailed instructions. **Make sure that all information entered here is included in the Methods section of the manuscript.**	N/A

**Format for specific study types**<br><br>**Human Subject Research (involving human participants and/or tissue)**<br>• Give the name of the institutional review board or ethics committee that approved the study<br>• Include the approval number and/or a statement indicating approval of this research<br>• Indicate the form of consent obtained (written/oral) or the reason that consent was not obtained (e.g. the data were analyzed anonymously)<br><br>**Animal Research (involving vertebrate animals, embryos or tissues)**<br>• Provide the name of the Institutional Animal Care and Use Committee (IACUC) or other relevant ethics board that reviewed the study protocol, and indicate whether they approved this research or granted a formal waiver of ethical approval<br>• Include an approval number if one was obtained<br>• If the study involved *non-human primates*, add *additional details* about animal welfare and steps taken to ameliorate suffering<br>• If anesthesia, euthanasia, or any kind of animal sacrifice is part of the study, include briefly which substances and/or methods were applied<br><br>**Field Research**<br><br>Include the following details if this study involves the collection of plant, animal, or other materials from a natural setting:<br>• Field permit number<br>• Name of the institution or relevant body that granted permission	
**Data Availability**<br><br>Authors are required to make all data underlying the findings described fully available, without restriction, and from the time of publication. PLOS allows rare exceptions to address legal and ethical concerns. See the PLOS Data Policy and FAQ for detailed information.	Yes - all data are fully available without restriction

A Data Availability Statement describing where the data can be found is required at submission. Your answers to this question constitute the Data Availability Statement and **will be published in the article**, if accepted.<br><br>**Important:** Stating 'data available on request from the author' is not sufficient. If your data are only available upon request, select 'No' for the first question and explain your exceptional situation in the text box.<br><br>Do the authors confirm that all data underlying the findings described in their manuscript are fully available without restriction?	
**Describe where the data may be found in full sentences. If you are copying our sample text, replace any instances of XXX with the appropriate details.**<br><br>• If the data are **held or will be held in a public repository**, include URLs, accession numbers or DOIs. If this information will only be available after acceptance, indicate this by ticking the box below. For example: *All XXX files are available from the XXX database (accession number(s) XXX, XXX.).*<br>• If the data are all contained **within the manuscript and/or Supporting Information files**, enter the following: *All relevant data are within the manuscript and its Supporting Information files.*<br>• If neither of these applies but you are able to provide **details of access elsewhere**, with or without limitations, please do so. For example:<br><br>*Data cannot be shared publicly because of [XXX]. Data are available from the XXX Institutional Data Access / Ethics Committee (contact via XXX) for researchers who meet the criteria for access to confidential data.*<br><br>*The data underlying the results presented in the study are available from (include the name of the third party*	The data is publicly available.

and contact information or URL).<br>• This text is appropriate if the data are owned by a third party and authors do not have permission to share the data.<br><br>* typeset	
Additional data availability information:	

Manuscript Click here to access/download;Manuscript;Manuscript.docx

Title page

Title **The extent of wealth poverty induced inequality in nutrition among children born during 2010-16 in India**

Author's Name Junaid Khan[1*] & Sanjay K Mohanty [2]

Authors' Affiliation [1]International Institute for Population Sciences,
Govandi Station Road, Deonar,
Mumbai-400088, India
E-mail address: statjun@gmail.com

[2] Professor, Department of Fertility Studies
International Institute for Population Sciences,
Govandi Station Road, Mumbai-400088,
Maharashtra, India
E-mail address: sanjayiips@yahoo.co.in

[*]Corresponding Author Junaid Khan
International Institute for Population Sciences,
Govandi Station Road, Mumbai-400088,
Maharashtra, India
E-mail address: statjun@gmail.com
Mobile Contact: +91-9653601397

Introduction

Though there are different forms of child malnutrition, undernutrition is much more prevalent in developing countries and stunting is the most severe form of undernutrition among the children and has the severe effect on child's survival, health and overall physical development [1, 2]. Due to household poverty, poor diet practice, poor environment and poor care during pregnancy, the foetal/child growth remains poor [1]. Stunting is the form of chronic malnutrition which is defined as the height-for-age z-score less than minus two standard deviation (-2SD) below the median of a reference standard population [3]. The measure of stunting identifies the shortness in achievable height among the children under age five for his/her age and informs about the nutritional history [4].

South Asia is home to 58.7 million stunted children and carries 40% of the global burden of stunting among the preschool children. The Global Nutrition Targets of the 2030 Sustainable Development Agenda aimed to reduce the burden of undernutrition by 40% by the end of 2025 [5]. India being the second most populous country, constitutes the largest share of the child population and stunted children worldwide [6]. Over the last two decades, India has shown a decrease (52% during 1992-93 to 38% during 2015-16) in the prevalence of child stunting but the variation is high across the states and districts of India [7, 8]. With a national average of 38%, the prevalence of stunting children is lowest (20%) in Kerala and highest (48%) in Bihar whereas the sub population of India conceals a large variation. Previous studies show detrimental evidences on child stunting and its association with the socio-economic characteristics[9-11]. The global burden of stunting prevalence is such acute that still one in four pre-school children suffer from stunting and almost 15% of the total deaths among under five children is attributable to stunting [12, 13].

India still stands as the development paradox and in terms of overall human development index (HDI) ranks 130 globally though it has made an improvement in terms of absolute reduction in poverty, increase in life expectancy and improvement in education and standard of living but failed in terms of social progress in the last decade [14-17]. However, the inequality in developmental parameters like

health, education and health care access are large within the country [18, 19]. At the same time it is also evident that it is the poor with low income show poor health status and malnourishment among those children from the poor families[20, 21].

Previous studies showed that nutritional status among the children under age five in the developing countries varies largely across the sub population and household's socioeconomic characteristics largely determines the nutritional status among the children [12, 22-24]. Thus, studies exploring the inequalities in population health are important to find the health gap across the heterogeneous population within a particular country or across countries. And the inequality in health could be due to economic wellbeing or due to social gap [25, 26]. Most of the previous studies based in developing country settings focused on poverty induced inequality in population health. A large number of studies examined the distribution of income and its association with the health parameters and suggested higher income difference leads to lower standards of population health [27, 28]. A Systematic review of studies reported increase in the number of studies measuring health inequalities in different domains of population health like - mortality, communicable and non-communicable diseases, nutrition, mental health, risk factors and injuries where wealth status and income is commonly used as the measure of equity followed by educational attainment and gender bias[29]. A number of studies examined the related socio-economic inequality specific to child health parameters and used regression based decomposition to measure the wealth gap in child health [30-32]. In this direction, this study adds to the existing knowledge in the domain of wealth based inequality in child stunting using the most recent round of National Family Health Survey for India. It used the Erreygers corrected measure of concentration indices to measure the concentration of stunting prevalence across the population sub groups subject to wealth poverty and decomposed the concentration indices by socio-demographic characteristics and across states using Gonzalo-Almorox and Urbanos-Garrido method.

Methods

Data

We utilised the data of children aged 0-59 months from the National Family Health Survey (NFHS)-2015-16. NFHS is one of the important surveys in India which provides necessary information on child health, most importantly child nutrition-stunting, underweight and wasting along with other health and household indicators and the survey estimates are most commonly used among the policy makers and among the government and non-governmental institutions to introduce new health intervention in the population. NFHS, 2015-16 is the fourth round in the series of DHS in India and all the NFHSs rounds contribute to the demographic database for India.

Sample Size and sampling

NFHS, 2015-16 survey adopted a multistage sampling strategy and used the 2011 Census India sampling frame. The villages in the rural areas and census enumeration blocks in the urban areas served as the primary sampling units (PSUs) for the first stage of sample selection. A total of 28,586 PSUs were selected randomly across the country and the fieldwork was completed in 28,522 clusters. Households within the PSUs served as the second stage sampling units. PSUs with fewer than 40 households were merged to the nearest PSU. Using probability proportional to size sampling, villages were selected from each rural stratum and in the urban areas, census enumeration blocks (CEBs) were selected from the list of CEBs,obtained from the Office of the Registrar General and Census Commissioner; New Delhi. Prior to the main survey, a complete household mapping and listing was done in the selected rural and urban PSUs. And within the selected PSUs (consisting of 300 or more households), households were divided into segments of 100-150 households. And finally, two of the segments were selected using systematic sampling with probability proportional to segment size.

Variables and Measurements

Outcome variable

The main outcome variable of the study is stunting which is an anthropometric measure of child's nutritional status. To measure the nutritional status among the children, Children's height (length for children under age 2 years), weight and age in months were used and the anthropometric measure of child's nutritional status i.e., length-for- age (LFA) Z-score for children under age 2 years and height-for- age (HFA) Z-score for those children above age 2 years were generated. To define stunting among the study children, the WHO, 2006 child growth standard was referred for both LFA as well as HFA Z scores. As both the scores LFA and HFA help to identify the impaired linear growth among the children under age two and above two respectively, we used the term stunting commonly throughout the study. During the survey of NFHS-4, Infantometer (Seca 417) was used to measure the recumbent length of the children under age two years and Stadiometer (Seca 213) was used to measure the height of the children aged 24-59 months. Seca 874 scale was used to measure the weight of the children.

Operational Definition

Wealth Poverty: NFHS collected the asset based (consumer goods like owning of television, bicycle, radio etc.) information from the randomly selected households and provided the wealth index as a proxy measure on household's economic wellbeing [33]. A set of 37 asset based indictors were used in this round of NFHS to create the wealth score for the households using a principal component analysis (PCA). And any member of the same household was assigned the same status of economic wellbeing, the household belongs to. The five economic classes of wealth quintiles are- poorest, poorer, middle, richer & richest.

Correlates

Child's individual characteristics, maternal characteristics and the household's characteristics are considered as the potential correlates for child stunting. Table 1 depicts the variables included in this

study along with their categories. Child's characteristics include socio demographic and economic characteristics, while community- level variables include the common characteristics of study subjects in an enumeration area such as region and place of residence. The description of the correlates is as follows- **Age of the child:** age of the child is captured in months and categorised as 0-5, 6-11, 12-23, 24-35, 36-47 & 48-59 months. **Sex of the child:** Sex of the child (male/female) is a dichotomous variable and a bio-demographic identification of the child. **Birth order of the child:** birth order of a particular child is categorised into four categories as- 1, 2-3, 4-5 & 6+. **Immunization status of the child:** child's immunization status is a key variable which signifies the utilization of health care services for vaccination by parents for their children. Child's immunization status has been compiled from the data which has been categorised as –no, partial and full immunization. **Mother's educational attainment:** mother's educational attainment has been categorised into four categories and are as follows- no education, primary completed, secondary completed and higher educated. **Mother's stature:** Maternal stature has been directly utilised as an indicator of maternal anthropometry and has been categorised in a dichotomous way. Mother's with less than 145 cm of stature are considered as stunted and otherwise. **Residence:** Place of residence of the children is another binary demographic variable and the categories are rural/urban. **Toilet facility:** As per the definition of NFHS-4, access to toilet facility has been categorised as improved and unimproved toilet facility. **Drinking water facility:** Similar to toilet facility, access to drinking water has also been categorised following the definition of NFHS-4 and households with access to any of these sources- piped water, public tap/standpipe, tube well or borehole, protected dug well, protected spring, rainwater, community RO (Reverse Osmosis) plant are classified as improved source of drinking water and unimproved otherwise. **Regions:** As per the NFHS definition, states are also grouped into six different regions namely, North, Central, East, North-East, South and West.

Statistical Analyses

A total of 225,002 children aged 0-59 months were analysed in this study. Taking care of the survey design, all the estimates provided are weighted.

Of the total 259,627 cases there were 23,172 cases for which the anthropometric information was not available. For the rest of the children, there are 10,235 cases found to be flagged, 21 cases for which the age in days is out of the plausible limits and there are 1,197 cases for which the height information is found to be out of the plausible limits.

The standard measure of socio-economic inequality for any health indicator for a particular population [34, 35] is given by the following-

$$Concentration\ Index\ (CI) = \frac{2}{\mu}\ cov\ (\gamma_i, R_i) \ldots (1)$$

Where μ is the mean of stunted children, $\gamma_i$is the nutritional status of the i-th children and R_i is the cumulative percentage that the i-th child represents over the total population once the i-th child ranked by socio-economic status here in this study by wealth score. The value of concentration index ranges between -1 to +1. A negative value implies that stunting is more concentrated among the wealth poor population while a positive value indicates that stunting is more concentrated among the rich population. And the value zero denotes the perfect example of no wealth related inequality in the health measure of the children.

Here in this study we do not use the conventional measure of concentration index to measure the socio-economic inequality rather we introduce the measure Erreygers' corrected concentration index taking care of the binary nature of the child's nutritional status variable[36]. The expression of the Erreygers' corrected concentration is given as following-

$$E(y) = \frac{4\mu}{y^{max} - y^{min}} CI \ldots (2)$$

Where μ is the mean prevalence of stunting and y^{max} and y^{min} are the two extreme values. To decompose the concentration indices of a binary variable subject to wealth poverty we utilised the generalised linear modelling (GLM) of a binomial family with probit link function which is recommended and provides the consistent estimate of the effects of demographic and socio-economic factors on child stunting independent of the choice of reference category[37, 38]. As we utilised the

Erreygers corrected concentration index to measure the inequality, we employed the Gonzalo-Almorox and Urbanos-Garrido method [39] of decomposing and the expression is given as below-

$$E(y) = 4.\sum_j (\beta_j^k \bar{x}_j)\, CI_j + GCI_\varepsilon \; \ldots(3)$$

Where E(y) is the Erreygers corrected concentration index, $\bar{x}_j$ is the mean of the j-th explanatory variable, CI_j is the average value of the j-th variable, β_j^k is the partial effect for the k-th category of the j-th variable and GCI_ε is the generalised concentration index of the error term. The decomposition of CI provides the estimates on elasticity, absolute contribution and percentage contribution in the overall inequality. Statistical analysis is performed using STATA version 14.1 (StataCorp™, Texas).

Results

Descriptive statistics

The dataset is complete in terms of all the concerned variables included in the analyses. Table 1 shows description of the children included in the study. Among the study children, 20% belong to the 12-23 month age group with lowest (8%) in the 0-5 month age group. The mean age of the children is 30 months. Fifty-two percent of the total children are male. Almost two-fifth (37%) of the study children are of first birth order. Only, half of the children are fully immunized and one-tenth of the children did not receive any of the doses of full immunization. Mothers to 31% of the children have no formal education. Twelve percent of the children's mothers are found to be less than 145cm in height. Seventy six percent of the children are rural and half of the children do not have access to improved sanitation facility. Twelve percent of the children do not have the access to safe drinking water. Around 41 percent of the study children belong to one of the socially excluded groups-scheduled caste or scheduled tribe. Half of the children belong to the lowest two wealth quintiles (Poorest and Poorer).

Distribution of HAZ scores

Box plots are drawn for the each of the explanatory variables to understand the distributional characteristics of the HAZ score as well as skewness and data quartiles of the scores given the categories of selected variables. The line that divides the box into two parts shows the mid-point of the data which is the median value of the z-score. The z-scores of half of the children population fall below the median z-score. Fig1-Fig11 visualises the HAZ scores for all the concerned variables. Fig 1 shows the distribution of the z-score by age groups which shows the shape of the boxes is almost uniform and short indicating a less variation in the score within every age group of children. Fig2 depicts the pattern of HAZ score by birth order of the children and the boxes are not very short which suggests that children within a particular group of the defined sub population (birth order) have quite different z-scores among them. This hints about the differential of nutritional status among the children of a specific birth order. It is also observed that with increasing birth order, the HAZ score for higher number of children falls below the score -200. The shape of the box for full immunization is observed shorter than those children with no immunization (Fig3). By mother's education, the HAZ scores of the children are observed to vary in different ranges and higher the educational attainment of the mothers, the median value is also found to be increasing (Fig4). Fig 5 shows that lower the stature of mothers, children show lower HAZ scores among them. Similarly, Fig6 describes the regional distribution of HAZ scores among the children. Children from the Central and Eastern part of India show low HAZ scores with higher percentage of children scoring below the -200 HAZ score. Similarly rest of the figures explain the nature of HAZ score among the children by residence, sex, toilet facility, access to improved drinking water and wealth quintile.

Table 2 provides the estimated prevalence of stunting by wealth quintile and by background characteristics. These estimates provide the pattern of stunting prevalence among the heterogeneous child population subject to wealth poverty. Within each of the wealth stratum, there remains large variation in the stunting prevalence by child's population characteristics- age of children, birth order, mother's education, mother's height and across regions. At the national level, wealth poverty does show a clear distinct differential in the stunting prevalence and children from the poorest wealth

quintile show the highest prevalence (51%) with a gradual decrease over the higher wealth quintiles. Children of age 24-39 months carry the highest prevalence than rest of the children and among them children from the poorest wealth quintile show comparatively higher burden of stunting. Sex of the child does not show a substantial difference in stunting burden across the wealth quintiles. At the national level, children with no immunization show higher stunting level (42%) followed by full immunization and partial immunization. Mother's education shows a clear gradient in terms of the stunting prevalence within every stratum of wealth quintiles. Subject to wealth poverty children of the no educated mothers carry the higher burden of stunting across India. It is observed that children of mothers with no education and from the poorest wealth quintile carry quite high prevalence of stunting (55%). Mother's anthropometry shows a large difference in the burden of child stunting and the prevalence is as high as 57% among the children of the stunted mothers (height less than 145 cm). With a sharp difference within wealth quintiles, it is observed that the burden of child stunting is double among the children of stunted mothers from the richest quintile than their counterpart from the same quintile. Though there is a rural-urban gap evident at the national level but within wealth quintiles, no steep gap has been observed over the wealth quintiles. Access to improved sanitation shows a varying level of stunting burden across the wealth quintiles and access to improved sanitation does show a lower burden of stunting in different wealth quintiles and nationally. Access to safe drinking water does not show much variation in the stunting prevalence. Regionally, the wealth pattern of child stunting is not very uniform although the poorest wealth quintile from all the regions show a very high burden of stunting with the Central region carrying a prevalence of 54%. Table 3 shows the wealth pattern of stunting burden across the states of India. There are states where the sample size is quite low when estimated within different wealth strata.

Concentration of stunting among heterogeneous child population

Table 4 gives the estimates of concentration indices measuring the wealth based concentration of stunting by background characteristics of the children. This gives a detailed understanding of inequality in child nutrition, the occurrence of stunting among the heterogeneous child population in terms of wealth poverty. We observed statistically significant concentration indices among the

different sub-population of children. It is observed that concentration of the stunted children is higher among the wealth poor within the sub-population. Age of the child, birth order of the child, immunization status, mother's education, mother's anthropometry (height), access to safe drinking water and across the regions the variation in inequality is large. Among the different age groups of children, children of age 36-47 months show the largest inequality (ECI: -0.28; 95% CI: -.294, -.265) followed by those children in the 24-35 months and 48-59 months. Higher concentration of stunting is observed among the higher age group of children across India. Though there is no much difference observed by sex but the inequality is quite high among both male and female children. Analysing the wealth inequality among the children of different birth orders, it is found that children of first birth order (ECI: -.217; 95% CI: -.228,-.206) show the highest concentration of stunting and it is lowest among six and higher ordered births. Children's immunization status also shows a varying level of concentration of stunting. Due to wealth poverty the concentration of stunting is found lowest among the fully immunised children (ECI: -.254; 95% CI: -.264, -.244). Mother's educational attainment does show a varying concentration of stunting prevalence and children of higher educated mothers carry a lower concentration of stunting prevalence within them. Mother's anthropometric status- whether stunting or not shows a difference in the concentration of stunted children and with the exposure to higher wealth poverty the concentration of stunted children is observed more among those children whose mother are not stunted. Access to improved sanitation and safe drinking water shows higher concentration of stunted children when exposed to wealth poverty. The regional pattern of concentration indices show that Eastern region of India shows highest concentration (-.215) of stunted children followed by North East India, Central India and Western India. The state pattern of concentration indices are shown in Table 5. Table 5 demonstrates the wealth disparity within states and the associated concentration of stunted children within a specific state.

Decomposition of the concentration Indices

Results of decomposition of the concentration indices are shown in Table 6 & 7. Table 6 shows the decomposition of CIs by background characteristics and state specific decomposition of the CIs are shown in Table 7.

The Erreygers concentration index for a category of a particular variable gives the pro-rich/pro-poor distribution of stunting within that subgroup of children subject to wealth distribution and decomposing the concentration indices, we estimated the elasticity, absolute contributions and percentage of contribution in the overall inequality for each of the factors under study. The measure of elasticity in this case gives the sensitivity to occurrence of stunting within a subgroup of children from the reference group subject to wealth poverty. And the sign indicates the likelihood to stunting among the children with a certain characteristics. In this study we found the estimated concentration indices to be fairly negative suggesting a concentrated distribution of stunting prevalence among the children of a certain characteristics with higher wealth poverty.

Factors like child age, birth order and sanitation show positive elasticity with the CIs being negative in some cases. All the other determinants of child stunting show a negative value. Among the children of different age groups, children in the age group of 12-23, 24-35 and 36-47 months show the highest elasticity within the range of 0.049-0.051. This shows that children of these age groups are more likely to be stunted with higher wealth poverty compared the reference group of children. The absolute contribution of wealth poverty on stunting are found to be negative for the last two age groups of children. For the rest of the children, the positive absolute contribution suggests that unequal distribution of stunted children in these age groups will decrease if wealth is uniformly distributed. Though the absolute contribution of sex is almost negligible yet the corresponding elasticity is found negative for the female child compared the male child. This indicates that due to wealth disparity, female child are less likely to be stunted than the male child. The respective elasticity for the children of different birth orders has been found positive which indicates that due to wealth poverty higher ordered births are more likely to be stunted than the first ordered births. Immunization status of the children does show a varying burden of stunting and with a negative elasticity (-0.005), fully immunized children are less likely to be stunted. All the categories of mother’s education show negative elasticity suggesting lower likelihood of stunting among children compared the reference group of children. Mother’s education as a determinant of child stunting solely explains 33% of the overall inequality due to uneven distribution of wealth. In other way

educational attainment shows a pro-rich distribution signifying the fact that mothers from the wealthy households are higher educated and children of higher educated mothers carry the lower prevalence of stunting. Mother’s anthropometric status also explains almost 8% of the overall inequality in child stunting given the wealth disparity. And child whose mother are not stunted are less likely to be stunted due to pro-poor distribution of wealth poverty. Urban children are less likely to be stunted than the rural children and place of residence as a covariate explains 5% of the overall inequality. Children from those households with no access to improved sanitation show more likelihood to stunting compared to those who have access to improved sanitation. Sanitation as a factor explains 24% of the overall inequality in stunting among Indian children. Whereas drinking water does not show any absolute contribution to the overall inequality. Among the different regions of India, North-East and Southern part of India show a negative elasticity which suggests that children from these two regions are less likely to be stunted than those children from the Northern part of India. Regional classification of the children thus explained a total of 6% of the overall inequality.

As per the study framework, all the factors explain 76% of the overall inequality in stunting among Indian children under age five. The unexplained part suggests the effect of unobserved heterogeneity influencing the distribution of those stunted children in the heterogeneous child population of India subject to unequal distribution of wealth poverty.

Discussion

This study assessed the wealth based inequality in child stunting using Errygers corrected concentration indices (CIs) and decomposed the CIs to estimate the elasticity, absolute contribution and percentage of contribution for each of the covariates demonstrating the inequality in child’s nutritional status that prevails across the states and sub-population of the children considering the global accountability of wealth disparity in India. As per the recent estimates by National Health Family Survey, 2015-16 it is quite evident that the children in India carry a persistently higher prevalence of stunting though poverty reduction and reduction in child malnutrition remained in top among the public health agendas in India. Though there are nutrition specific interventions running

across the country still poverty differential in child nutrition is acute and children from certain population groups are more vulnerable subject to the household's economic wellbeing. Chronic poverty situation in India plays a major role to determine child's nutritional health [40, 41]. And In India, still two-fifth of all the children under age five is stunted and the prevalence of stunting varies largely across the heterogeneous child population of India.

From this study, it is evident that prevalence of stunting among Indian children shows a concentrated distribution with critically higher burden in some of the population groups. It is apparent that rich-poor gap in stunting prevalence is quite stark and more than half of the children from the poorest wealth quintile are stunted nationally. And within the poorest section, children of stunted mothers carry critically higher burden of stunting. The prevalence of child stunting is lopsided very high in the lower wealth quintiles across states and among children of different socio-demographic characteristics. Among the different factors, age of children, birth order, mother's educational attainment, mother's anthropometry (height), place of residence, sanitation (access to improved toilet facility) and the region a child where he/she belongs to are the key factors explaining the gap in stunting across the wealth quintiles. From the age pattern of inequality it is clear that children in the age group of 36-47 and 48-59 months carry higher inequality in terms of nutritional status. Result for the gender pattern shows a diminished differential in stunting prevalence in the presence of persisting wealth inequality. With a negligible absolute contribution in the overall inequality, sex of the child does not show any strong evidence on gender based inequality in nutrition given the wealth poverty situation in India.

The concentration indices explained the unequal distribution of stunted children within population subgroups and across states of India. The estimates of CIs based on the most recent data shows a statistically significant and negative concentration index nationally and the pattern remained consistently high and negative among all the subgroups (ranging in between 0.06 to 0.28) and across states (ranging in between .02 to 0.25) suggesting a pro-poor distribution of the stunted children. Eventually, the wealth based inequality was found highest in Gujarat among all the states/union territories with higher concentration of stunted children along the poorest wealth quintiles with Uttar

Pradesh and Assam being the second and third highest in terms the wealth based inequality in child nutrition. The decomposition of the concentration indices demonstrated the wealth poverty driven inequalities among the children across the subgroups and across the states. Given the prevailing wealth disparity across India, it is found that socio-demographic determinants of child malnutrition put a disproportionate burden of stunting and each of the factors contributes differently to the overall inequality in child malnutrition. In this regard, mother's educational attainment, sanitation condition, mother's stature and place of residence of the children play a dominant role to contribute to the overall inequality in child malnutrition.

This particular study demonstrated the evidence on poverty based inequality in stunting prevalence across the heterogeneous child population of India using wealth as a measure of economic wellbeing of the household than income or consumption expenditure of the household. Though across the surveys, the measure of economic wellbeing varies, there are certain merits and demerits associated with each of the measures. Arguably, a direct measurement of household's total income is a good measure of money metric poverty and the purchasing capacity of a household. While the measure of consumption expenditure of a household provides direct information on expenditure incurred on food items as well as the calorie consumed. On the other hand, wealth based measure is quite an indirect measure as well as a proxy measure to economic wellbeing [33, 42]. Like other standard, Demographic Health Surveys, NFHS also collected the wealth based information on an exhaustive range set of asset based indicators and provides the standardised measure of wealth based economic wellbeing of a household which is extensively used in this study to capture the poverty induced nutritional inequality among Indian children.

This study adds to the existing knowledge of nutritional inequality among under-five children and the uneven distribution of stunting burden among the heterogeneous child population subject to wealth poverty using the most recent round of National Family Health Survey, 2015-16. In this regard, this study corroborates the most recent pattern of nutritional inequality and identifies the sub-group of children vulnerable to malnourishment with higher concentration of stunted children. Thus it is global to mention that poverty being a major driver of child malnutrition it needs the most careful attention

to alleviate poverty and to make those households food secure from the lowest wealth quintiles to uplift the nutritional health of the children.

The limitation of this study is that this is a cross sectional study and this fails to draw a casual inference between wealth poverty and child stunting rather this study is specific to examine the concentration of stunted children across the sub population with a major thrust to identify the related inequality in child stunting due to wealth poverty across India. State specific measurement of inequality is also explored within the state-specific sample. Potentially, this study estimated the Erryger's corrected concentration indices and decomposed it to measure the elasticity and the associated contribution in the overall inequality of a particular factor which has not been attempted previously.

Conclusion

This study assessed how socioeconomic inequality in child nutrition is persistent in India given the wealth poverty situation across the subpopulation and across states. This study also examined how different socioeconomic factors contribute to the overall inequality and found that wealth poverty contributes positively in terms of the socio-economic inequality in stunting among children in India. Though poverty reduction is one of the SDG goals implemented in India, this study reinforces the importance of poverty alleviation which may help to reduce the overall inequality in child nutrition parallel to improvement in terms of the other health outcomes in the population. It is also found that there are several factors like, mother's education, sanitation condition, mother's anthropometrical height and place of residence plays a strong role in the overall inequality. Thus careful focus and implementation of appropriate interventions to improve mother's education, sanitation in the rural areas and monitoring mother's nutritional health along with taking care of the diet and food pattern among the young women can reflect on their stature which in turn helps to reduce stunting among children in India.

Abbreviations

NFHS: National Family Health Survey; CI: Concentration index; HAZ: Height-for-Age z score; LFA: Length-for- Age; SD: Standard Deviation; HDI: Human Development Index; DHS: Demographic Health Survey; MOHFW: Ministry of Health and Family Welfare; PSU: Primary Sampling Unit; CEB: Census Enumeration Block; PCA: Principal Component Analysis; RO: Reverse Osmosis; GLM: Generalised Linear Modelling; SC: Scheduled Caste; ST: Scheduled Tribe; ECI: Erreygers Concentration Index; ICDS: Integrated Child Development Service; UNDP: United Nations Development Programme; NRHM: National Rural Health Mission; MRD: Ministry of Rural Development

Declarations

Ethics approval and consent to participate

As the study is based on secondary data available in public domain for research; no ethical approval was required from any institutional review board (IRB).

Consent for Publication

Not applicable

Availability of data and material

This study is based upon National Family Health Survey (NFHS), 2015-16 data and is publicly available through DHS data repository and could be accessed upon a data request. National Family Health Survey data set is available at https://dhsprogram.com/

Competing interests

On behalf of all authors, the corresponding author states that there is no conflict of interest.

Funding

Authors did not receive any grant or funding from any source to conduct this study.

Author's contributions

JK & SKS apprehended and conceptualized the study. JK reviewed the literature, performed the analysis and prepared the draft of the manuscript. SKM critically edited and revised the

manuscript. This study is part of JK's PhD dissertation work. All the authors read and approved the final manuscript.

Acknowledgements

Not applicable

References

1. Wild CP, Miller JD, Groopman JD. Mycotoxin control in low-and middle-income countries: International Agency for Research on Cancer Lyon, France; 2015.
2. Jonah CM, Sambu WC, May JD. A comparative analysis of socioeconomic inequities in stunting: a case of three middle-income African countries. Archives of Public Health. 2018;76(1):77.
3. Organization WH. WHO child growth standards: length/height-for-age, weight-for-age, weight-for-length, weight-for-height and body mass index-for-age: methods and development. 2006.
4. Organization WH. Catalogue of health indicators: a selection of important health indicators recommended by WHO programmes. Geneva: World Health Organization, 1996.
5. UNICEF. Levels and trends in child malnutrition UNICEF-WHO-World Bank Group joint child malnutrition estimates: key findings of the 2015 edition. New York: UNICEF, WHO, World Bank Group. 2015.
6. Sri J. Children in India 2012–A Statistical Appraisal. New Delhi: Social Statistics Division. 2012:24.
7. Sciences IIfP. India: National Family Health Survey 1992-93: IIPS; 1995.
8. Sciences IIfP, ICF. National Family Health Survey (NFHS-4), 2015–16: India. IIPS Mumbai; 2017.
9. Fenske N, Burns J, Hothorn T, Rehfuess EA. Understanding child stunting in India: a comprehensive analysis of socio-economic, nutritional and environmental determinants using additive quantile regression. PloS one. 2013;8(11):e78692.
10. Rizal MF, van Doorslaer E. Explaining the fall of socioeconomic inequality in childhood stunting in Indonesia. SSM-population health. 2019;9:100469.
11. Galgamuwa LS, Iddawela D, Dharmaratne SD, Galgamuwa G. Nutritional status and correlated socio-economic factors among preschool and school children in plantation communities, Sri Lanka. BMC public health. 2017;17(1):377.
12. De Onis M, Blössner M, Borghi E. Prevalence and trends of stunting among pre-school children, 1990–2020. Public health nutrition. 2012;15(1):142-8.
13. Black R, Allen L, Bhutta Z, Caulfield L, de Onis M, Ezzati M, et al. Maternal and Child Undernutrition Study Group: Maternal and child undernutrition 1-maternal and child undernutrition: global and regional exposures and health consequences. Lancet. 2008;371:243-60.
14. Conceição P. Human Development Report 2019, Beyond income, beyond averages, beyond today: inequalities in human development in the 21st century. United Nations development Programme. 2019;1.
15. Chandy L, Gertz G. Poverty in numbers: The changing state of global poverty from 2005 to 2015: Brookings Institution Washington, DC; 2011.
16. Chen S, Ravallion M. The developing world is poorer than we thought, but no less successful in the fight against poverty. The Quarterly Journal of Economics. 2010;125(4):1577-625.
17. Ghani E. The poor half billion in South Asia. HALF BILLION. 2010:1.
18. Sen A. The possibility of social choice. American Economic Review. 1999;89(3):349-78.

19. Alkire S, Santos ME. Acute multidimensional poverty: A new index for developing countries. United Nations development programme human development report office background paper. 2010;(2010/11).
20. Varadharajan KS, Thomas T, Kurpad AV. Poverty and the state of nutrition in India. Asia Pacific journal of clinical nutrition. 2013;22(3):326.
21. Nelson M. Childhood nutrition and poverty. Proceedings of the nutrition society. 2000;59(2):307-15.
22. Fotso J-C. Child health inequities in developing countries: differences across urban and rural areas. International journal for equity in health. 2006;5(1):9.
23. Fotso J. Urban–rural differentials in child malnutrition in Sub-Saharan Africa: Trends and socioeconomic correlates. Health Place. 2007;13:205-23.
24. Gwatkin DR, Rutstein S, Johnson K, Pande R, Wagstaff A. Socio-economic differences in health, nutrition, and population. Washington, DC: World Bank. 2000.
25. Moss NE. Social Inequalities and Health. Project HOPE-The People-to-People Health Foundation, Inc.; 1995.
26. van Deurzen I, Van Oorschot W, van Ingen E. The link between inequality and population health in low and middle income countries: policy myth or social reality? PloS one. 2014;9(12):e115109.
27. Lynch J, Smith GD, Harper SA, Hillemeier M, Ross N, Kaplan GA, et al. Is income inequality a determinant of population health? Part 1. A systematic review. The Milbank Quarterly. 2004;82(1):5-99.
28. Wilkinson RG, Pickett KE. Income inequality and population health: a review and explanation of the evidence. Social science & medicine. 2006;62(7):1768-84.
29. Bhan N, Rao KD, Kachwaha S. Health inequalities research in India: a review of trends and themes in the literature since the 1990s. International journal for equity in health. 2016;15(1):1-8.
30. Chalasani S. Understanding wealth-based inequalities in child health in India: a decomposition approach. Social science & medicine. 2012;75(12):2160-9.
31. Kessels R, Erreygers G. A direct regression approach to decomposing socioeconomic inequality of health. Health Economics. 2019;28(7):884-905.
32. KIA AA, Goodarzi S, Asadi H, Khosravi A, Rezapour A. A Decomposition Analysis of Inequality in Malnutrition among Under-Five Children in Iran: Findings from Multiple Indicator Demographic and Health Survey, 2010. Iranian Journal of Public Health. 2019;48(4):748.
33. Rutstein SO, Johnson K. The DHS wealth index. DHS comparative reports no. 6. Calverton: ORC Macro. 2004.
34. Wagstaff A, Paci P, Van Doorslaer E. On the measurement of inequalities in health. Social science & medicine. 1991;33(5):545-57.
35. Kakwani N. On a class of poverty measures. Econometrica: Journal of the Econometric Society. 1980:437-46.
36. Erreygers G. Correcting the concentration index. Journal of health economics. 2009;28(2):504-15.
37. Doorslaer Ev, Koolman X. Explaining the differences in income-related health inequalities across European countries. Health economics. 2004;13(7):609-28.
38. Yiengprugsawan V, Lim LL, Carmichael GA, Dear KB, Sleigh AC. Decomposing socioeconomic inequality for binary health outcomes: an improved estimation that does not vary by choice of reference group. BMC research notes. 2010;3(1):57.
39. Gonzalo-Almorox E, Urbanos-Garrido RM. Decomposing socio-economic inequalities in leisure-time physical inactivity: the case of Spanish children. International journal for equity in health. 2016;15(1):106.
40. Humphries DL, Dearden KA, Crookston BT, Woldehanna T, Penny ME, Behrman JR. Household food group expenditure patterns are associated with child anthropometry at ages 5, 8 and 12 years in Ethiopia, India, Peru and Vietnam. Economics & Human Biology. 2017;26:30-41.

41. Harttgen K, Klasen S, Vollmer S. Economic growth and child undernutrition in sub-Saharan Africa. Population and development review. 2013;39(3):397-412.
42. Rutstein SO. The DHS wealth index: approaches for rural and urban areas. DHS working papers, 2008.

Fig1. Box plot of HAZ score by age of the child, NFHS, 2015-16, India

Fig2. Box plot of HAZ score by birth order of the child, NFHS, 2015-16, India

Fig3. Box plot of HAZ score by immunization status of the child, NFHS, 2015-16, India

Fig4. Box plot of HAZ score by mother's education of the child, NFHS, 2015-16, India

Fig5. Box plot of HAZ score by mother's height of the child, NFHS, 2015-16, India

Fig6. Box plot of HAZ score across regions, NFHS, 2015-16, India

Fig7. Box plot of HAZ score by place of residence, NFHS, 2015-16, India

Fig8. Box plot of HAZ score by sex of the child, NFHS, 2015-16, India

Fig9. Box plot of HAZ score by sanitation facility, NFHS, 2015-16, India

Fig10. Box plot of HAZ score by source of drinking water, NFHS, 2015-16, India

Fig11. Box plot of HAZ score by wealth quintile, NFHS, 2015-16, India

Table1: Frequency distribution of children under five years, India, 2015-16

**Variables**	[illegible]stribution	**Variables**	Distribution
**Age in months**		**Wealth Quintile**	
0-5	8.4	Poorest	25.9
6-11	10.2	Poorer	23.6
12-23	20.1	Middle	20.1
24-35	20.0	Richer	16.8
36-47	21.1	Richest	13.6
48-59	20.1	**Regions**	
**Sex of the child**		North	18.9
Male	51.7	Central	29.3
Female	48.3	East	21.0
**Birth order**		North East	14.2
1	36.9	West	6.9
2-3	47.1	South	9.7
4-5	12.0	**N**	**2,25,002**
6+	4.0		
**Immunization**			
No	9.6		
Partial	40.2		
Full	50.3		
**Mother's Education**			
No education	30.7		
Primary completed	14.6		
Secondary Completed	45.4		
Higher	9.3		
**Mother's height**			
<145cm	11.6		
>=145 cm	88.4		
**Residence**			
Rural	76.2		
Urban	23.8		
**Sanitation**			
Improved	50.2		
Unimproved	49.8		
**Drinking water**			
Improved	87.5		
Unimproved	12.5		

Table 2: Prevalence of stunting among under-five children by wealth poverty and by background characteristics, India, 2015-16

Variables	Poorest	Poorer	Middle	Richer	Richest	Total
**Age in months**						
0-5	23.2(4852)	21.5(4672)	18.1(3754)	18.3(3077)	15.7(2568)	19.9(18923)
6-11	31.6(5734)	25.3(5444)	21.5(4680)	17.6(3917)	14.2(3191)	23.0(22966)
12-23	55.0(11465)	48.2(10464)	41.2(9281)	34.1(7807)	26.6(6272)	42.7(45289)
24-35	58.2(11397)	49.0(10658)	41.1(9150)	31.9(7750)	24.0(6141)	42.7(45096)
36-47	59.0(12583)	49.6(11280)	40.9(9369)	31.0(7789)	24.4(6389)	43.2(47410)
48-59	54.5(12299)	45.9(10644)	37.9(8902)	29.5(7475)	20.3(5998)	39.9(45318)
**Sex of the child**						
Male	52.0(29716)	44.1(27224)	36.8(23564)	29.9(19534)	23.6(16322)	38.8(116360)
Female	51.0(28614)	42.9(25938)	36.1(21572)	28.4(18281)	20.6(14237)	37.9(108642)
**Birth order**						
1	48.6(15222)	40.0(18054)	33.3(17768)	26.5(16726)	20.4(15276)	33.3(83046)
2-3	50.3(26746)	44.0(25256)	37.6(21909)	30.6(18079)	23.7(14022)	38.9(106012)
4-5	54.7(11625)	48.9(7346)	43.3(4329)	37.8(2524)	28.4(1130)	48.9(26954)
6+	59.8(4737)	52.1(2506)	45.3(1130)	38.5(486)	36.5(131)	55.0(8990)
**Immunization**						
No	51.0(8481)	44.1(5699)	37.2(3615)	29.5(2377)	24.3(1306)	42.4(21478)
Partial	48.6(25538)	40.1(22191)	33.9(17636)	28.6(14315)	21.0(10725)	36.7(90405)
Full	54.7(24311)	46.2(25272)	38.4(23885)	29.6(21123)	22.8(18528)	39.1(113119)
**Mother's Education**						
No education	55.0(35877)	49.3(18466)	44.0(9171)	39.6(4196)	37.9(1268)	50.8(68978)
Primary completed	49.2(10051)	44.8(10470)	40.9(6993)	35.4(3929)	30.2(1392)	43.4(32835)
Secondary Completed	43.2(12058)	38.8(23034)	33.9(26434)	28.2(24446)	23.7(16219)	32.8(102191)
Higher	39.5(344)	30.9(1192)	24.9(2538)	22.1(5244)	18.0(11680)	20.8(20998)
**Mother's height**						
<145cm	64.6(10230)	58.2(7166)	52.3(4598)	45.8(2776)	39.7(1367)	57.0(26137)
>=145 cm	48.5(47958)	41.1(45869)	34.6(40430)	27.7(34948)	21.3(29111)	35.8(198316)
**Residence**						
Rural	51.6(55722)	43.3(47553)	35.9(35012)	28.4(21557)	21.8(11675)	41.2(171519)
Urban	49.8(2608)	44.8(5609)	38.2(10124)	30.0(16258)	22.4(18884)	31.0(53483)
**Sanitation**						
Improved	47.0(4683)	41.1(16920)	35.9(26240)	29.0(31168)	22.4(28194)	30.9(107205)
Unimproved	52.3(51119)	45.0(33545)	38.0(16597)	31.9(4523)	20.3(639)	46.4(106423)
**Drinking water**						
Improved	52.3(47074)	44.2(43140)	37.1(37630)	29.4(32305)	22.4(26836)	38.9(186985)
Unimproved	48.6(8728)	39.9(7325)	33.9(5207)	29.5(3386)	21.7(1997)	36.8(26643)
**Regions**						
North	49.9(4567)	43.0(7863)	37.2(9130)	31.4(9331)	24.3(11590)	34.4(42481)
Central	53.7(21565)	48.2(15861)	41.9(11207)	35.5(9184)	25.2(8150)	44.4(65967)
East	51.1(22938)	41.3(11921)	32.8(6739)	24.0(4007)	17.7(1715)	42.0(47320)
North East	46.9(6295)	37.6(10610)	28.6(7944)	18.9(4926)	16.1(2230)	35.0(32005)
West	49.0(1947)	44.9(3227)	39.7(3767)	30.5(3624)	22.4(2853)	35.5(15418)
South	43.8(1018)	39.6(3680)	33.0(6349)	25.9(6743)	18.3(4021)	29.3(21811)
**India**	**51.5(58330)**	**43.5(53162)**	**36.5(45136)**	**29.2(37815)**	**22.2(30559)**	**38.4(225002)**

Numbers within parentheses

Table3: Prevalence of stunting among under-five children by wealth poverty and by states/UTs, India, 2015-16*

	Wealth Quintiles											
**States/UTs**	Poorest	N	Poorer	N	Middle	N	Richer	N	Richest	N	Total	N
Andaman And Nicobar Islands	56	37	35	72	31	171	18	198	14	100	23	578
Andhra Pradesh	40	126	42	473	34	941	26	712	17	347	31	2,599
Arunachal Pradesh	36	937	33	1,066	28	961	21	653	19	234	29	3,851
Assam	48	2,725	38	3,459	27	1,563	18	825	14	283	36	8,855
Bihar	55	12,330	47	5,418	36	2,594	28	1,500	22	433	48	22,275
Chhattisgarh	43	3,095	41	1,922	39	1,299	29	955	24	959	38	8,230
Dadra & Nagar Haveli	52	71	49	60	41	49	32	62	27	35	42	277
Daman & Diu	NA	NA	30	26	26	50	24	133	18	120	23	329
Goa	NA	NA	39	33	37	46	22	118	12	178	20	378
Gujarat	53	909	49	1,493	44	1,558	32	1,347	22	1,137	38	6,444
Haryana	58	200	49	636	39	1,273	35	1,877	26	2,889	34	6,875
Himachal Pradesh	38	47	41	282	32	602	26	915	17	679	26	2,525
Jammu & Kashmir	41	825	35	1,894	28	1,863	24	1,355	18	1,156	28	7,093
Jharkhand	52	5,348	46	2,328	41	1,409	29	876	20	546	46	10,507
Karnataka	51	444	44	1,468	39	2,005	32	1,674	23	717	36	6,308
Kerala	NA	NA	32	57	26	324	22	879	16	961	20	2,235
Lakshadweep	NA	NA	NA	NA	NA	NA	26	149	26	114	27	281
Madhya Pradesh	49	7,456	45	5,043	41	3,342	36	2,883	27	2,548	42	21,272
Maharashtra	47	964	42	1,615	37	2,064	30	1,964	23	1,383	34	7,990
Manipur	44	716	34	1,768	27	1,503	21	879	17	390	29	5,256
Meghalaya	50	625	47	1,520	44	1,194	28	365	27	119	44	3,823
Mizoram	43	334	42	689	31	1,138	23	1,283	19	865	28	4,309
Nagaland	40	710	31	1,513	27	890	18	472	12	240	29	3,825
Delhi	97	NA	44	46	40	150	35	270	27	674	32	1,145
Odisha	44	3,916	35	2,538	25	1,771	20	1,007	16	496	34	9,728
Puducherry	38	NA	24	73	20	156	28	312	22	396	24	945
Punjab	52	50	44	207	31	673	29	1,150	21	2,666	26	4,746
Rajasthan	50	3,151	44	3,720	39	3,124	32	2,578	27	2,343	39	14,916
Sikkim	32	NA	37	90	34	459	23	286	22	54	29	898
Tamil Nadu	36	229	35	1,102	31	2,182	24	2,241	19	1,082	27	6,836
Tripura	39	239	28	505	15	236	11	163	12	45	24	1,188
Uttar Pradesh	57	11,014	50	8,896	43	6,566	36	5,346	25	4,643	46	36,465
Uttarakhand	48	286	40	1,069	41	1,425	30	1,151	22	1,076	34	5,007
West Bengal	41	1,344	35	1,637	32	965	21	624	15	240	33	4,810
Telangana	45	159	39	434	30	554	24	578	15	304	28	2,029
**India**	**51**	**58,330**	**43**	**53,162**	**36**	**45,136**	**29**	**37,815**	**22**	**30,559**	**38**	**2,25,002**

*Chandigarh has been dropped due to smaller sample size. NA: less than 25 sample points

Table4: Concentration index of child stunting by background characteristics, India, 2015-16

Variables	No. of obs.	Index value	95% CI		Robust std. error	p-value
Age in months						
0-5	18,923	-0.058	-0.077	-0.039	0.010	0.00
6-11	22,966	-0.135	-0.152	-0.118	0.009	0.00
12-23	45,289	-0.221	-0.236	-0.205	0.008	0.00
24-35	45,096	-0.269	-0.284	-0.254	0.008	0.00
36-47	47,410	-0.280	-0.294	-0.265	0.007	0.00
48-59	45,318	-0.266	-0.280	-0.252	0.007	0.00
**Sex of the child**						
Male	1,16,360	-0.225	-0.234	-0.215	0.005	0.00
Female	1,08,642	-0.235	-0.245	-0.225	0.005	0.00
**Birth order**						
1	83,046	-0.217	-0.228	-0.206	0.006	0.00
2-3	1,06,012	-0.204	-0.214	-0.194	0.005	0.00
4-5	26,954	-0.144	-0.162	-0.126	0.009	0.00
6+	8,990	-0.132	-0.160	-0.103	0.014	0.00
**Immunization**						
No	21,478	-0.193	-0.216	-0.171	0.011	0.00
Partial	90,405	-0.208	-0.218	-0.198	0.005	0.00
Full	1,13,119	-0.254	-0.264	-0.244	0.005	0.00
**Mother's Education**						
No education	68,978	-0.107	-0.118	-0.096	0.006	0.00
Primary completed	32,835	-0.114	-0.133	-0.095	0.010	0.00
Secondary Completed	1,02,191	-0.141	-0.152	-0.131	0.005	0.00
Higher	20,998	-0.078	-0.096	-0.060	0.009	0.00
**Mother's height**						
<145cm	26,137	-0.168	-0.190	-0.145	0.012	0.00
>=145 cm	1,98,316	-0.214	-0.222	-0.207	0.004	0.00
**Residence**						
Rural	1,71,519	-0.207	-0.214	-0.199	0.004	0.00
Urban	53,483	-0.182	-0.198	-0.166	0.008	0.00
**Sanitation**						
Improved	1,07,205	-0.158	-0.169	-0.147	0.006	0.00
Unimproved	1,06,423	-0.139	-0.149	-0.129	0.005	0.00
**Drinking water**						
Improved	1,86,985	-0.235	-0.243	-0.227	0.004	0.00
Unimproved	26,643	-0.202	-0.224	-0.180	0.011	0.00
Regions						
North	42,481	-0.190	-0.206	-0.174	0.008	0.00
Central	65,967	-0.203	-0.214	-0.192	0.006	0.00
East	47,320	-0.215	-0.228	-0.202	0.007	0.00
North East	32,005	-0.212	-0.232	-0.193	0.010	0.00
West	15,418	-0.203	-0.229	-0.176	0.014	0.00
South	21,811	-0.168	-0.188	-0.148	0.010	0.00
**India**	**2,25,002**	**-0.229**	**-0.237**	**-0.222**	**0.004**	**0.00**

[illegible]ble5: Concentration index of child stunting by states/UTs in India, NFHS, 2015-16

**States**	No. of obs.	CI value	95% CI		Robust std. error	p-value
Andaman & Nicobar Islands	578	-0.217	-0.313	-0.121	0.049	0.00
Andhra Pradesh	2,599	-0.167	-0.211	-0.123	0.023	0.00
Arunachal Pradesh	3,851	-0.127	-0.170	-0.085	0.022	0.00
Assam	8,855	-0.222	-0.248	-0.197	0.013	0.00
Bihar	22,275	-0.175	-0.191	-0.159	0.008	0.00
Chandigarh	174	-0.155	-0.278	-0.031	0.063	0.02
Chhattisgarh	8,230	-0.134	-0.162	-0.105	0.015	0.00
Dadra & Nagar Haveli	277	-0.210	-0.360	-0.061	0.076	0.01
Daman & Diu	329	-0.080	-0.197	0.037	0.060	0.18
Goa	378	-0.194	-0.286	-0.102	0.047	0.00
Gujarat	6,444	-0.247	-0.281	-0.214	0.017	0.00
Haryana	6,875	-0.165	-0.197	-0.132	0.017	0.00
Himachal Pradesh	2,525	-0.167	-0.217	-0.117	0.025	0.00
Jammu & Kashmir	7,093	-0.165	-0.197	-0.133	0.016	0.00
Jharkhand	10,507	-0.179	-0.204	-0.154	0.013	0.00
Karnataka	6,308	-0.170	-0.216	-0.125	0.023	0.00
Kerala	2,235	-0.081	-0.119	-0.044	0.019	0.00
Lakshadweep	281	-0.023	-0.129	0.083	0.054	0.67
Madhya Pradesh	21,272	-0.152	-0.171	-0.133	0.010	0.00
Maharashtra	7,990	-0.176	-0.213	-0.140	0.019	0.00
Manipur	5,256	-0.172	-0.204	-0.140	0.016	0.00
Meghalaya	3,823	-0.116	-0.160	-0.073	0.022	0.00
Mizoram	4,309	-0.184	-0.232	-0.135	0.025	0.00
Nagaland	3,825	-0.167	-0.207	-0.128	0.020	0.00
Delhi	1,145	-0.131	-0.283	0.021	0.078	0.09
Odisha	9,728	-0.210	-0.236	-0.185	0.013	0.00
Puducherry	945	-0.007	-0.084	0.070	0.039	0.86
Punjab	4,746	-0.113	-0.146	-0.081	0.017	0.00
Rajasthan	14,916	-0.180	-0.203	-0.158	0.012	0.00
Sikkim	898	-0.115	-0.184	-0.047	0.035	0.00
Tamil Nadu	6,836	-0.120	-0.154	-0.086	0.017	0.00
Tripura	1,188	-0.211	-0.271	-0.152	0.030	0.00
Uttar Pradesh	36,465	-0.234	-0.248	-0.220	0.007	0.00
Uttarakhand	5,007	-0.173	-0.211	-0.136	0.019	0.00
West Bengal	4,810	-0.162	-0.199	-0.126	0.019	0.00
Telangana	2,029	-0.194	-0.250	-0.138	0.029	0.00
**India**	**2,25,002**	**-0.229**	**-0.237**	**-0.222**	**0.004**	**0.00**

[illegible]ble 6: Decomposition of concentration indices of stunting by background characteristics, India, 2015-16

	Stunting			
Variables	Elasticity	CI	Absolute contribution	% Contribution
Age in months				
0-5	base	base	base	base
6-11	0.004	0.005	0.000	-0.038
12-23	0.050	0.004	0.001	-0.317
24-35	0.049	0.011	0.002	-0.900
36-47	0.051	-0.005	-0.001	0.415
48-59	0.041	-0.010	-0.002	0.729
**Total**				**-0.111**
**Sex of the child**				
Male	base	base	base	base
Female	-0.007	-0.009	0.000	-0.105
**Total**				**-0.105**
**Birth order**				
1	base	base	base	base
2-3	0.007	0.004	0.000	-0.042
4-5	0.001	-0.309	-0.002	0.684
6+	0.001	-0.450	-0.002	0.893
**Total**				**1.535**
**Immunization**				
No	base	base	base	base
Partial	0.001	-0.032	0.000	0.051
Full	-0.005	0.063	-0.001	0.557
**Total**				**0.608**
**Mother's Education**				
No education	base	base	base	base
Primary completed	-0.005	-0.173	0.004	-1.598
Secondary Completed	-0.044	0.174	-0.031	13.421
Higher	-0.020	0.608	-0.048	20.910
**Total**				**32.733**
**Mother's height**				
<145cm	base	base	base	base
>=145 cm	-0.144	0.031	-0.018	7.645
**Total**				**7.645**
Residence				
Rural	base	base	base	base
Urban	-0.006	0.446	-0.010	4.498
**Total**				**4.498**
**Sanitation**				
Improved	base	base	base	base
Unimproved	0.037	-0.372	-0.055	23.760
**Total**				**23.760**
**Drinking water**				
Improved	base	base	base	base
Unimproved	-0.002	-0.019	0.000	-0.049
**Total**				**-0.049**
**Regions**				
North	base	base	base	base
Central	0.013	-0.121	-0.006	2.690
East	0.003	-0.317	-0.004	1.717
North East	-0.001	-0.172	0.000	-0.171
West	0.003	0.224	0.002	-1.042
South	-0.005	0.303	-0.006	2.729
**Total**				**5.924**

Table 7: Decomposition of concentration indices of stunting by states, India, 2015-16

	Stunting			
**States**	Elasticity	CI	Absolute contribution	% Contribution
Andaman & Nicobar Islands	0.000	0.373	0.000	-0.005
Andhra Pradesh	0.005	0.235	0.005	-1.974
Arunachal Pradesh	0.000	-0.055	0.000	0.009
Assam	0.005	-0.232	-0.004	1.842
Bihar	0.038	-0.428	-0.065	28.144
Chandigarh	0.000	0.644	0.000	-0.073
Chhattisgarh	0.005	-0.129	-0.003	1.086
Dadra & Nagar Haveli	0.000	-0.017	0.000	0.002
Daman & Diu	0.000	0.434	0.000	-0.003
Goa	0.000	0.563	0.000	0.000
Gujarat	0.009	0.214	0.008	-3.258
Haryana	0.004	0.481	0.007	-3.065
Himachal Pradesh	0.000	0.434	0.001	-0.266
Jammu & Kashmir	0.001	0.190	0.001	-0.274
Jharkhand	0.008	-0.334	-0.011	4.736
Karnataka	0.008	0.224	0.007	-2.961
Kerala	base	base	base	base
Lakshadweep	0.000	0.630	0.000	-0.004
Madhya Pradesh	0.016	-0.132	-0.009	3.753
Maharashtra	0.014	0.226	0.012	-5.346
Manipur	0.000	0.052	0.000	-0.019
Meghalaya	0.001	-0.118	0.000	0.160
Mizoram	0.000	0.325	0.000	-0.046
Nagaland	0.000	-0.077	0.000	0.019
Delhi	0.002	0.587	0.004	-1.740
Odisha	0.005	-0.245	-0.005	2.264
Puducherry	0.000	0.467	0.000	-0.033
Punjab	0.001	0.617	0.003	-1.263
Rajasthan	0.012	0.031	0.002	-0.676
Sikkim	0.000	0.283	0.000	-0.013
Tamil Nadu	0.005	0.317	0.006	-2.722
Tripura	0.000	-0.112	0.000	0.023
Uttar Pradesh	0.050	-0.115	-0.023	10.006
Uttarakhand	0.001	0.275	0.001	-0.613
West Bengal	0.010	-0.133	-0.005	2.262
Telangana	0.003	0.285	0.003	-1.317

Figure

Click here to access/download;Figure;Figure.docx

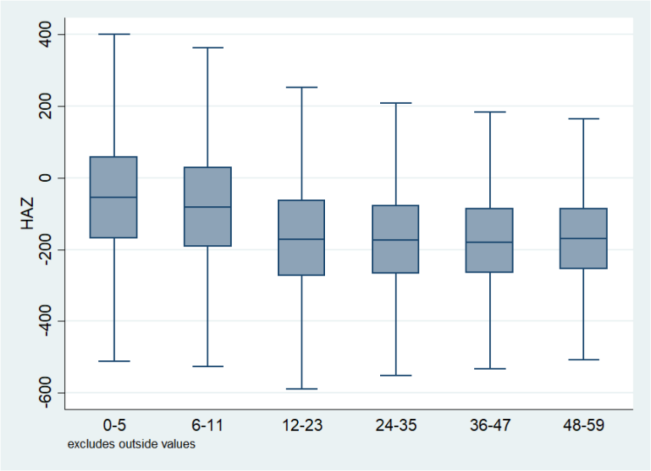

Fig1.

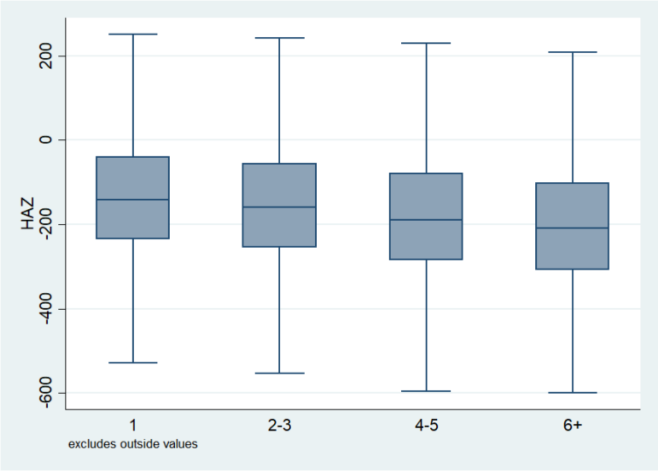

Fig2.

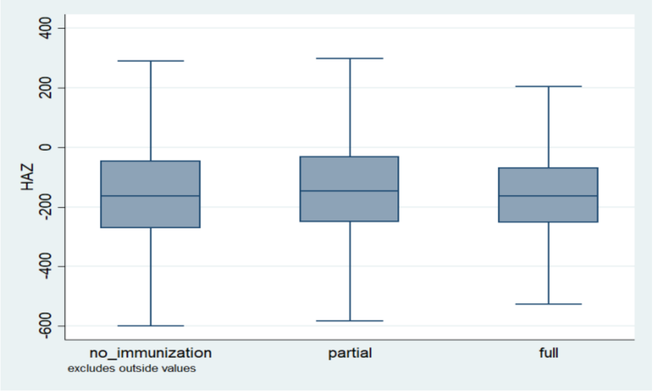

Fig3.

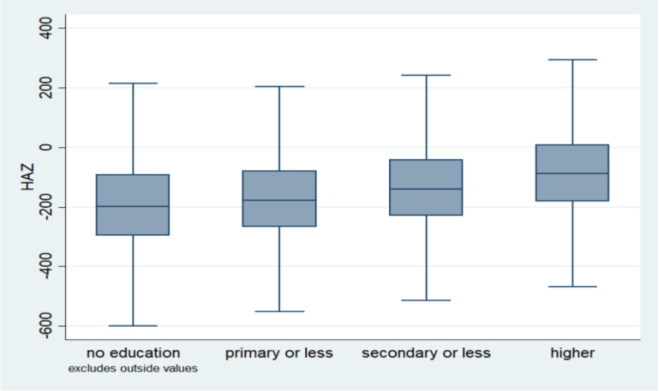

Fig4.

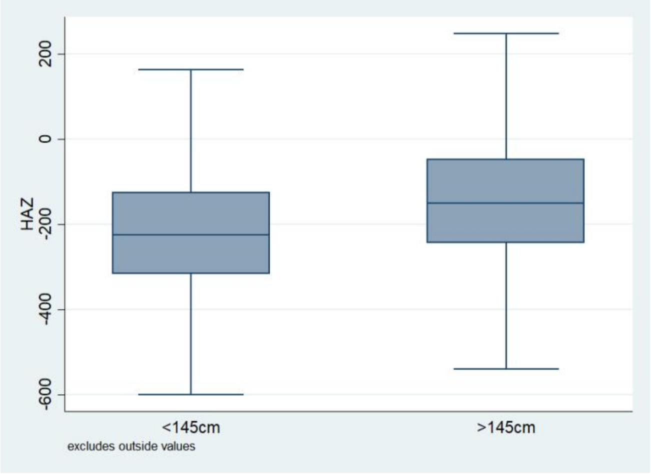

Fig5.

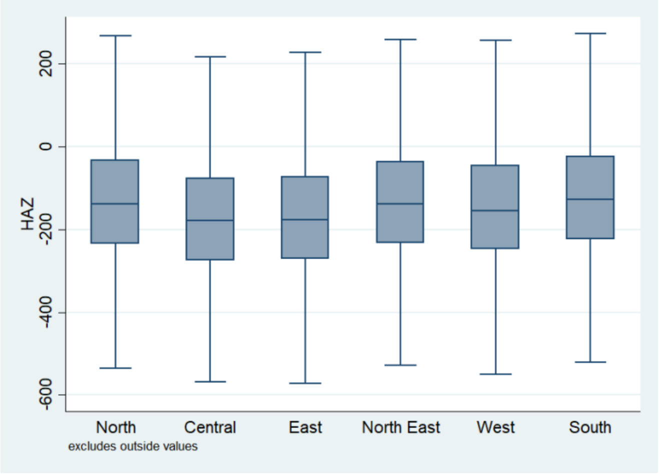

Fig6.

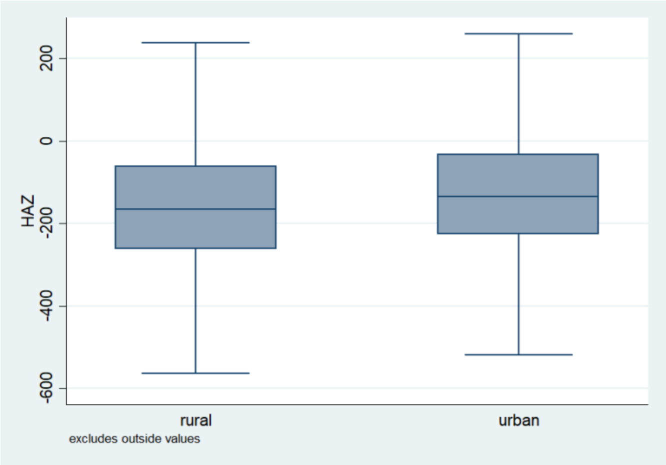

Fig7.

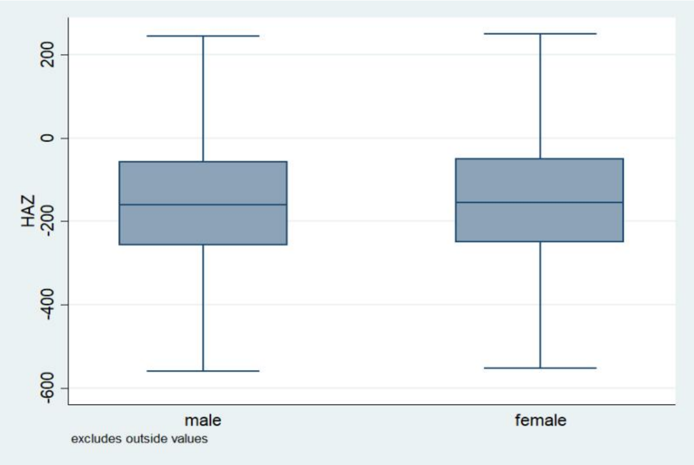

Fig8.

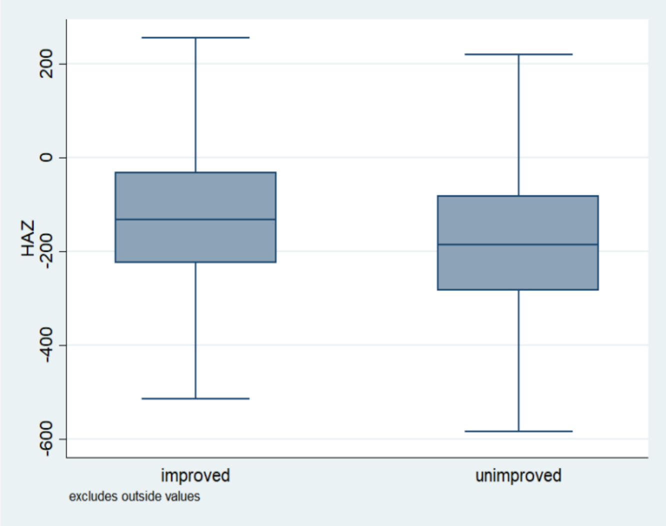

Fig9.

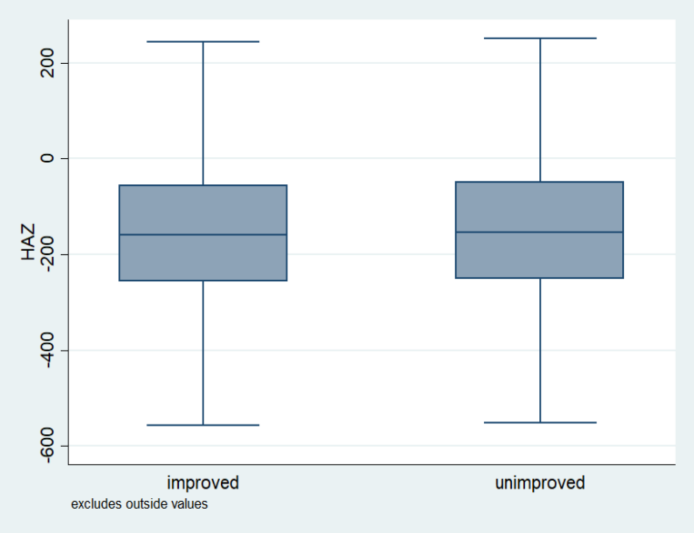

Fig10.

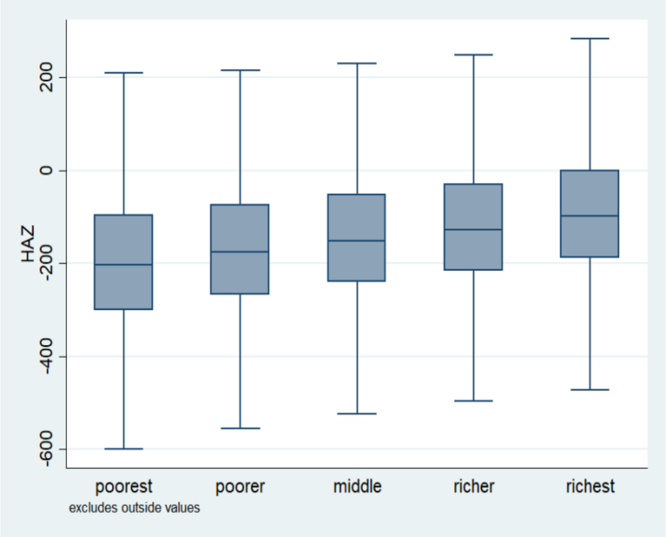

Fig11.
